# Mitochondrial metabolic reprogramming-mediated immunogenic cell death reveals immune and prognostic features of clear cell renal cell carcinoma

**DOI:** 10.3389/fonc.2023.1146657

**Published:** 2023-05-05

**Authors:** Lin Yang, Jing Xiong, Sheng Li, Xiaoqiang Liu, Wen Deng, Weipeng Liu, Bin Fu

**Affiliations:** Department of Urology, First Affiliated Hospital of Nanchang University, Nanchang, China

**Keywords:** mitochondrial metabolic reprogramming, immunogenic cell death, clear cell renal cell carcinoma, TME, immunotherapy

## Abstract

**Background:**

Mitochondrial metabolic reprogramming (MMR)-mediated immunogenic cell death (ICD) is closely related to the tumor microenvironment (TME). Our purpose was to reveal the TME characteristics of clear cell renal cell carcinoma (ccRCC) by using them.

**Methods:**

Target genes were obtained by intersecting ccRCC differentially expressed genes (DEGs, tumor VS normal) with MMR and ICD-related genes. For the risk model, univariate COX regression and K-M survival analysis were used to identify genes most associated with overall survival (OS). Differences in the TME, function, tumor mutational load (TMB), and microsatellite instability (MSI) between high and low-risk groups were subsequently compared. Using risk scores and clinical variables, a nomogram was constructed. Predictive performance was evaluated by calibration plots and receiver operating characteristics (ROC).

**Results:**

We screened 140 DEGs, including 12 prognostic genes for the construction of risk models. We found that the immune score, immune cell infiltration abundance, and TMB and MSI scores were higher in the high-risk group. Thus, high-risk populations would benefit more from immunotherapy. We also identified the three genes (*CENPA, TIMP1, and MYCN*) as potential therapeutic targets, of which *MYCN* is a novel biomarker. Additionally, the nomogram performed well in both TCGA (1-year AUC=0.862) and E-MTAB-1980 cohorts (1-year AUC=0.909).

**Conclusions:**

Our model and nomogram allow accurate prediction of patients’ prognoses and immunotherapy responses.

## Background

Renal cell carcinoma (RCC) is one of the most common urological malignancies, especially clear cell renal cell carcinoma (ccRCC), with approximately 431 288 new cases of RCC in 2020, 70% of which were ccRCC (1, 2). For non-metastatic RCC, surgical treatment can achieve curative results, but the high recurrence rate (40%) after surgery is a major factor affecting the long-term survival of patients (3). In addition, although systemic therapy is the standard of care for metastatic RCC, the long-term survival of patients with metastatic RCC has remained suboptimal for many years. With the rise of immunotherapy, combined treatment modalities targeting vascular endothelial growth factor and immune checkpoints offer new hope for improving the prognosis of metastatic RCC and high-risk limited RCC (4, 5). Nevertheless, the response rates and durability of treatments observed in clinical practice have not been satisfactory, mainly due to the lack of guiding and effective biomarkers (6, 7).

Metabolic reprogramming is the phenomenon that occurs when tumor cells undergo a rapid adaptive response to hypoxic and low oxygen conditions to proliferate and migrate in the tumor microenvironment (TME), and it is one of the important hallmarks of cancer (8). Mitochondria are known to play a key role in biosynthesis, energy metabolism, and signaling, and are dynamic organelles that coordinate functions such as metabolism, cell proliferation, and cell survival (9). Tumor cells are no exception, and their malignant transformation and development are dependent on mitochondrial energy metabolism. Interestingly, there is also metabolic heterogeneity in tumor tissues. The adaptive metabolic response of tumor cells not only regulates each other with the TME they are in but also influences the way genes are expressed, leading to great differences in the malignancy and therapeutic response of different individual tumors (10, 11).

For example, in ccRCC, lipid metabolism, and lactate metabolic processes, which are both closely related to the occurrence and progression of ccRCC, as well as the identification of potentially valuable therapeutic targets (*FBP1, HADH*, and *TYMP*) based on genes related to lactate metabolism by Sun et al. (11–14). With a deeper understanding of mitochondria in cancer development, cancer therapeutic modalities targeting mitochondrial metabolism are gradually being translated into clinical practice (15, 16). Surprisingly, recent studies have shown that mitochondrial metabolic reprogramming (MMR) can enhance immunogenic cell death (ICD) effects and thus optimize cancer immunotherapy outcomes (17, 18). ICD can activate antitumor immune effects by remodeling the TME. ICD stimulates dendritic cell (DC) maturation under endogenous conditions through the release of tumor-associated antigens and danger-associated molecular patterns, including adenosine triphosphate, high-motility group box 1, and calreticulin. Once DC mature, they deliver antigens to cytotoxic T lymphocytes, which then activate antitumor immunity (19, 20).

Therefore, our study aims to use MMR and ICD-related genes to identify and reveal clear cell renal cell TME and prognostic features to screen individuals suitable for immunotherapy to help clinicians in scientific decision-making.

## Materials and methods

### Datasets

We obtained the training dataset from the Cancer Genome Atlas (TCGA) database(https://portal.gdc.cancer.gov/). Including TCGA-KIRC TPM and raw counts gene expression data (tumor sample: 541, normal sample: 72) and clinical data of 533 patients. TCGA-KIRC data was processed by log2 (TPM+1) for subsequent analysis. Moreover, we excluded genes with too low expression (more than 25% of samples with counts expression less than 10). From the ArrayExpress database (https://www.ebi.ac.uk/biostudies/arrayexpress), E-MTAB-1980, represented the validation cohort, including 101 patients with ccRCC. The identification of MMR and ICD-related genes was based on the GeneCards database and previously published literature. To begin with, we searched the GeneCards database for “mitochondrial metabolic reprogramming” and “immunogenic cell death” and then screened for protein-coding genes with a correlation score greater than 0.5. We obtained 34 ICD-related genes from the results of Abhishek’s study (21). Eventually, we obtained 3103 genes related to MMR and 1732 genes related to ICD.

### Weighted gene co-expression network analysis (WGCNA)

The close association between MMR and ICD-related genes and ccRCC was confirmed using the R package “WGCNA”. Co-expression networks with scale-free topologies were selected using appropriate soft-threshold power. Further, to investigate gene connectivity in this network, a topological overlap matrix (TOM) was converted from the adjacency matrix. A hierarchical clustering tree (dendrogram) of genes was generated based on TOM, gene modules are represented by different branches on the clustering tree, and by different colors.

Clinical and immune features included stage, immune score, and CD8 T-cell infiltration levels. Each sample was assessed using the “estimate” package to determine its immune score, stromal score, estimate score, and tumor purity. The online analysis tool TIMER2.0 (http://timer.cistrome.org/) can download the immune cell infiltration score file for each TCGA-KIRC sample, and we used 22 immune cell infiltration scores based on the CIBERSORT algorithm.

### Identification and analysis of target genes (TGs)

Using the “DESeq2” package to screen the differentially expressed genes (DEGs) (tumor VS normal). A differential gene’s screening criteria require the p-value< 0.05, and the |log2fold change|> 1.5. The DEGs, MMR, and ICD-related genes were then intersected to obtain TGs for subsequent analysis.

Somatic mutation data of ccRCC samples were downloaded from the TCGA database, and the “Maftools” package was used to calculate the tumor mutational load (TMB) and visualize the mutation landscape of TGs. In addition, “clusterProfiler” was used for Gene Ontology (GO) and Kyoto Encyclopedia of Genes and Genomes (KEGG) analyses to understand which biological functions are associated with the TGs.

### Developing a risk model based on prognosis-related genes

Univariate COX regression and K-M survival analysis (screening criteria: p<0.01; GEPIA database, http://gepia.cancer-pku.cn/index.html) were performed using TCGA-KIRC expression and survival data to identify TGs most associated with overall survival (OS), which was then used to perform a least absolute shrinkage and selector operation analysis (LASSO) with the “glmnet” package. Patients’ risk scores were calculated by multiplying their expression value by their coefficient by summing the scores of each OS-related gene. The median risk score was used to distinguish low-risk patients from high-risk patients. In addition, principal component analysis (PCA) was used to assess how well model genes discriminated between samples. Further validating the model’s predictive capability, we analyzed TCGA-KIRC and E-MTAB-1980 cohorts using Kaplan-Meier (K-M) survival analyses. As well, we compared the OS and risk scores of patients with varying clinical characteristics.

### The immune landscape across different risk groups

For each sample, the TME score and infiltration level of 22 immune cells were calculated, and then the differences between the two risk groups were observed. Furthermore, immunoinhibitors and immunostimulators are closely related to the TME, we evaluated their expression levels in samples from different risk groups.

### Functional and gene set enrichment analysis (GSEA)

DEGs between the two risk groups were identified with the “limma” package. After using the “ClusterProfiler” package, GO and KEGG analyses were performed, and GSEA was to identify differential signaling pathways, biological effects, and gene sets that are enriched in different populations. The “GSVA” package was used to compute normalized enrichment scores for pathway and functional annotations using the gene set variation analysis (GSVA) approach. The “c2.cp.kegg.v7.4.symbols.gmt” annotation file was obtained from the MSigD. To further assess the immune-related functions score for each sample, we used the “GSEABase” and “GSVA” packages and then combined them with the immune gene set annotation file “immune. gmt” for enrichment analysis.

### The prediction of immunotherapy response and drug sensitivity

Palmeri’s study indicated that TMB and microsatellite instability (MSI) could predict the effect of immunotherapy (22). Therefore, we assessed the TMB levels and MSI status of the different risk groups. The TMB was calculated using the R package “maftools” and the microsatellite instability score was downloaded using the package “cBioPortalData” in the file “kirc_tcga _pan_can_atlas_2018”. We choose “MSI_SENSOR_SCORE”, if the score is greater than 0.3, it is defined as MSI, and vice versa as MSS. Furthermore, we used the “easier” package (23) to obtain the immune response score file and identify the relationship between risk score and immunotherapy response. Each sample’s sensitivity scores to varying drugs were calculated using the package “oncoPredict” (24), with higher scores indicating greater. The reference file “GDSC2_Expr (RMA Normalized and Log Transformed).rds” was from Cancer Drug Sensitivity Genomics (https://www.cancerrxgene.org/).

### Comprehensive analysis of model genes

Our subsequent analysis focused on the three genes with the highest absolute risk coefficients among the twelve model genes. The online analysis tools Gene Set Cancer Analysis (GSCA, http://bioinfo.life.hust.edu.cn/GSCA/#/) and TISIDB (http://cis.hku.hk/TISIDB/) were used to analyze the expression differences, methylation profiles, prognostic significance of these three genes in pan-cancer and immune relevance. In addition, we analyzed their distribution in different cell types using single-cell sequencing data from the online tool Tumor Immune Single-cell Hub 2 (TISCH2, http://tisch.comp-genomics.org/).

### Developing and validating nomograms

To screen for independent predictors of OS, univariate and multivariate COX regression analyses were performed for age, gender, stage, and risk score with a screening criterion of p<0.05. Nomograms were then constructed using the “ survival” and “ rms” packages. Furthermore, the model’s predictive performance was validated with calibration plots and receiver operating characteristics (ROC) analyses. The same analysis was performed for the E-MTAB-1980 cohort.

### Validation of gene expression and protein expressions

The real-time fluorescence quantitative polymerase chain reaction (RT-qPCR) was used to verify the expression levels of key genes. Human RCC cells are derived from the Procell Life Science&Technology Co., Ltd (Wuhan, China). After RNA was extracted using TRIzol reagent (Invitrogen, Thermo Fisher Scientific, Inc.), reverse transcription was performed using the Takara PrimeScript RT kit (Takara Bio, Inc., Otsu, Japan). Lastly, RT-qPCR was performed using the SYBR premix Ex Taq kit (Takara Bio, Inc., Otsu, Japan) on the Roche LightCycler96 real-time fluorescent quantitative PCR system. Based on the 2^−ΔΔCt method, the FC in mRNA was calculated.

### Statistical analysis

Our statistical analyses were performed using R (version 4.2.2) or GraphPad Prism (version 9.0). All statistics were two-sided tests, and p<0.05 was considered statistically significant if not otherwise stated. An analysis of Spearman correlation was performed to determine the correlation between two continuous variables. Comparing two independent samples with Wilcoxon rank sum tests and multiple samples with Kruskal-Wallis tests. Comparing two categorical variables was accomplished with the chi-square test. The significance of differences in K-M survival analysis was assessed using the log-rank test.

## Result

### Screening of TGs for subsequent analysis

**Figure 1:**
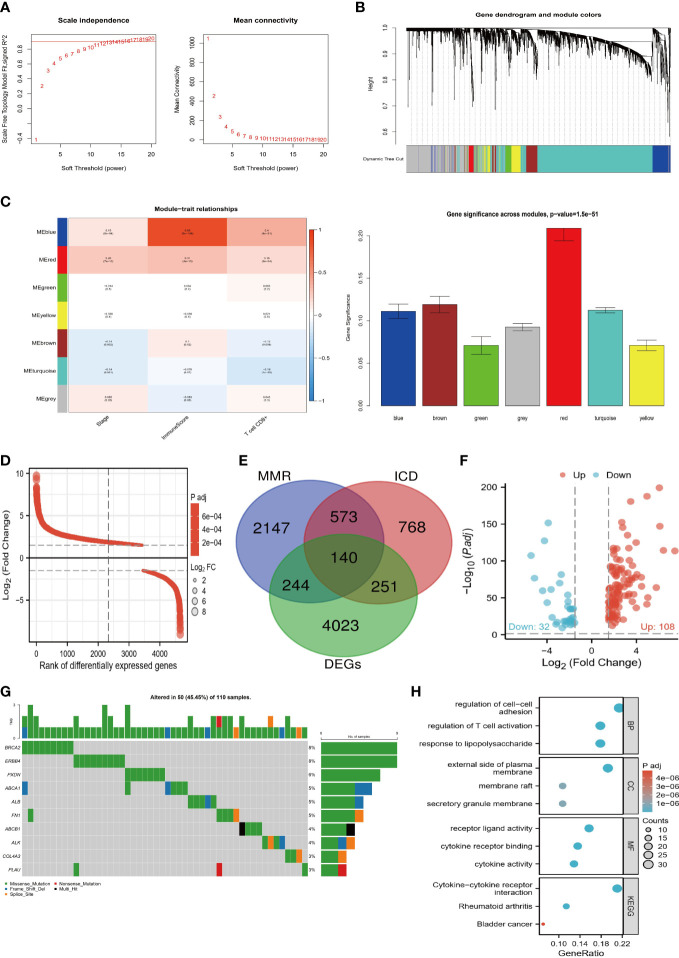
Screening process of target genes. **(A)** Determine the appropriate soft threshold power equal to 11. **(B)** Generate a hierarchical clustering tree of genes based on TOM, with different colors representing different modules. **(C)** The modules are closely related to the clinical features and TME of ccRCC, with the red module being the most important. **(D)** Differential ranking map of DEGs between tumor and normal tissues. **(E)** Venn diagram of DEGs, MMR, and ICD-related genes taking the intersection. **(F)** Volcano plot of 140 target genes. **(G)** Mutation waterfall plot of target genes. H Enrichment bubble map of biological functions and pathways of target genes.

Using the WGCNA algorithm, we evaluated the correlations of MMR and ICD-related genes with ccRCC prognosis and TME. As shown in **Figure 1A**, we chose 11 as the optimal soft threshold power to construct the co-expression network. Hierarchical clustering analysis was then performed based on weighted correlations, and these genes were classified into different modules (**Figure 1B**). **Figure 1C** shows that most of the modules are remarkedly related to the stage, immune infiltration score, and CD8 T cell infiltration abundance, with the red module being the most relevant. Hence, the development of ccRCC appears to be strongly linked to MMR and ICD-related genes. Therefore, we first screened the DEGs between tumor and normal tissues (**Figure 1D**) and then took the intersection of it and MMR and ICD-related genes to identify 140 target genes for subsequent analysis (**Figure 1E**). Comparatively to normal tissues, volcano map analysis showed that 108 genes had up-regulation and 32 genes had down-regulation (**Figure 1F**). In addition, the mutation landscape of TGs showed the highest frequency of mutations in BRCA2 and ERBB4 (**Figure 1G**). Their close association with immune-related functions and pathways was revealed by enrichment analysis, including regulation of T cell activation, cytokine receptor binding, cytokine activity, and Cytokine-cytokine receptor interaction (**Figure 1H**). This further corroborates that TGs are closely associated with the TME of ccRCC.

### Risk model based on 12 genes

**Figure 2:**
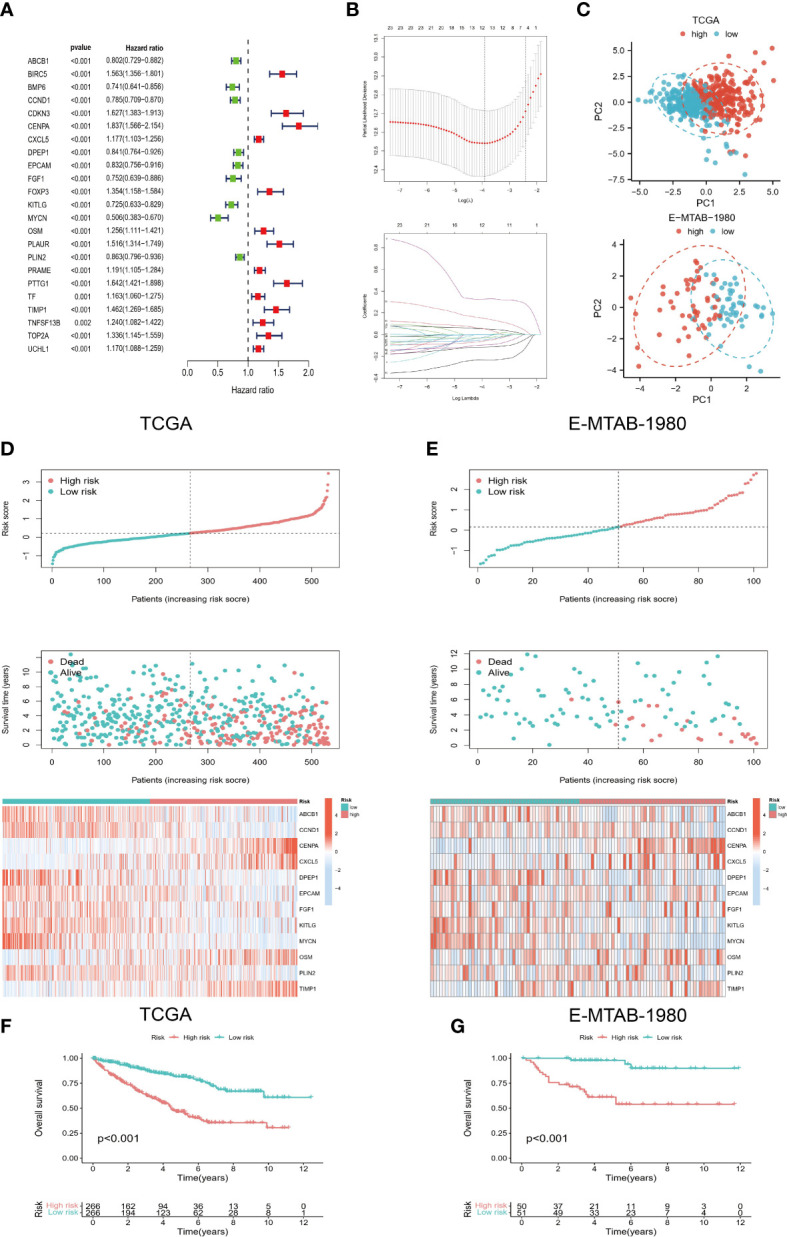
Development of risk model. **(A)** Prognostic genes were screened based on univariate COX regression and K-M survival analysis. **(B)** LASSO regression was used to construct a 12-gene-based risk model. **(C)** PCA analysis showed that the model genes had a good discriminatory ability. **(D, E)** Risk scores of TCGA and E-MTAB-1980 cohorts were correlated with survival status and model genes. **(F, G)** K-M survival analysis for high- and low-risk groups.

According to the screening criteria (p<0.01), 23 prognostic genes most associated with OS were identified (**Figure 2A**). On the basis of LASSO regression analysis, a 12-gene risk model was subsequently developed (**Figure 2B**) [risk score=ABCB1*(-0.114) + CCND1*(-0.002) +CENPA*0.328+CXCL5*0.053+DPEP1*(-0.058) +EPCAM*(-0.028) +FGF1*(-0.021) +KITLG*(-0.094) +MYCN*(-0.232) +OSM*0.052+PLIN2*(-0.047) +TIMP1*0.149]. The results of PCA also showed strong risk differentiation of model genes (**Figure 2C**). Furthermore, it can be observed from (**Figures 2D, E**) that the trends of risk scores and survival status of the training set TCGA and validation set E-MTAB-1980 are highly consistent. The heat map of the distribution of model genes with risk scores also remained almost consistent. The results of the K-M survival analysis also showed significant differences in OS between the two risk groups (**Figures 2F, G**), which indicates the strong predictive performance of our model.

### The risk score and clinical variables.

**Figure 3:**
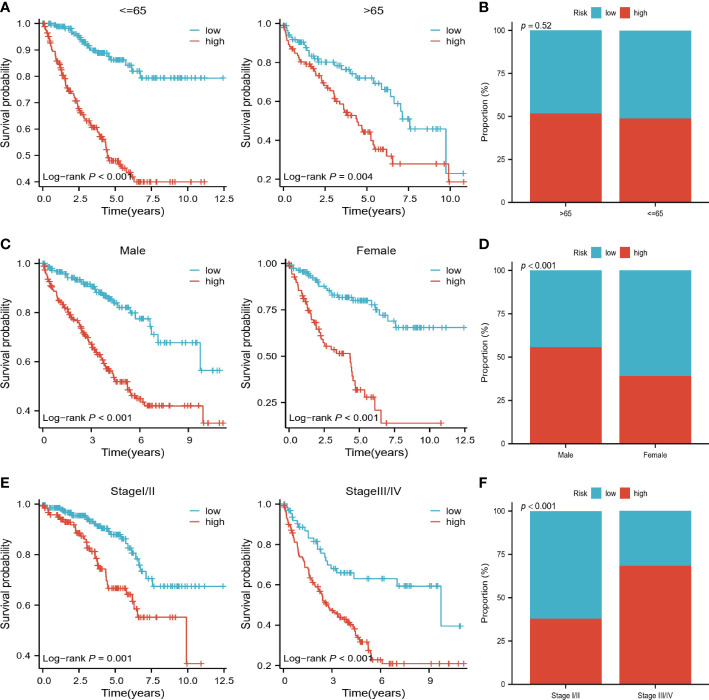
Relationship of risk scores to clinical variables. **(A, B)** K-M survival analysis and comparison of risk scores for high and low-risk groups in patients <=65 years and >65 years. **(C, D)** K-M survival analysis of high and low-risk groups of male and female patients with comparison of risk scores by gender. **(E, F)** K-M survival analysis of high- and low-risk groups of early and late-stage patients, with a comparison of risk scores by stage.

Depending on the patient’s age, gender, and stage, we classify them into different groups. It can be seen from **Figure 3A** that patients in the high-risk group had a worse prognosis regardless of age (<=65 years, >65 years). Risk scores between the two age groups did not differ significantly, however (**Figure 3B**). Patients with high-risk outcomes had shorter survival rates in both males and females, and males had a higher percentage of high-risk patients (**Figures 3C, D**). As well, both early (stage I/II) and late-stage (stage III/IV) patients in the high-risk group had a worse outcome, with a higher proportion of late-stage patients at high risk (**Figures 3E, F**).

### Immune landscapes in different risk groups

**Figure 4:**
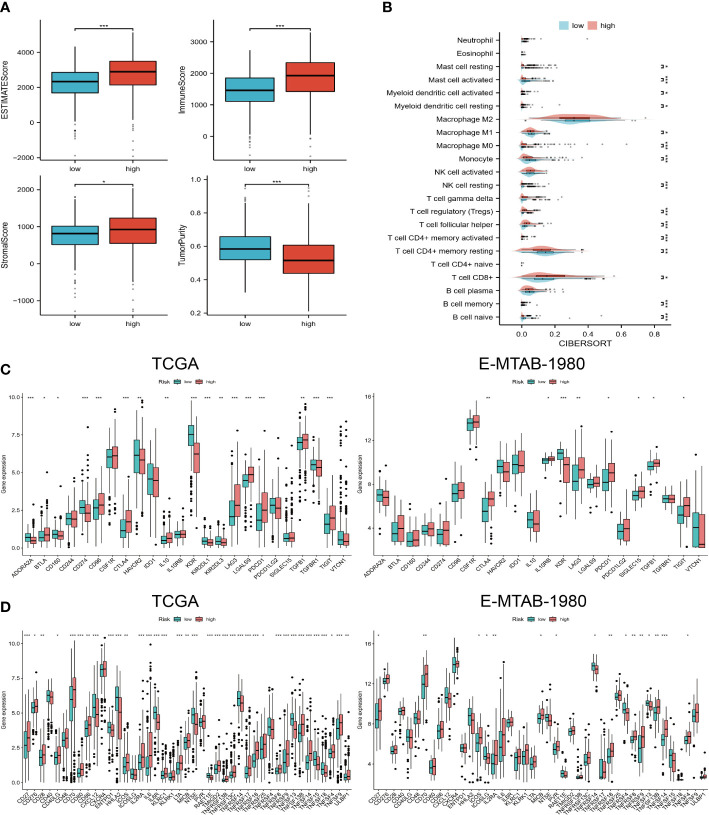
Immune landscapes in different risk groups. **(A)** Estimated scores, immune scores, stromal scores, and tumor purity for high- and low-risk groups. **(B)** 22 levels of immune cell infiltration. **(C, D)** Expression levels of immunoinhibitor and immunostimulator agents in TCGA and E-MTAB-1980 cohorts. “*”<0.5, "**" <0.01, and "***"<0.001.

In the high-risk group, we observed higher estimated scores, immune scores, and tumor purity scores (**Figure 4A**). Furthermore, higher levels of CD8+ T cells, TFH T cells, and memory-activated CD4+ T cells were found in the high-risk group (**Figure 4B**). There were also differences in immunoinhibitor and immunostimulator expression levels between the two risk groups. According to **Figure 4C**, high-risk individuals expressed higher levels of CTLA4, LAG3, PDCD1, TGFB1, and TIGIT. High-risk patients also expressed higher levels of immunostimulators, such as CD27, CD276, CD28, CD70, IL2RA, IL6, and TNFRSF18 (**Figure 4D**). In addition, we observed the same expression trend in the validation cohort.

### Functional and pathway differences between high and low-risk groups

**Figure 5:**
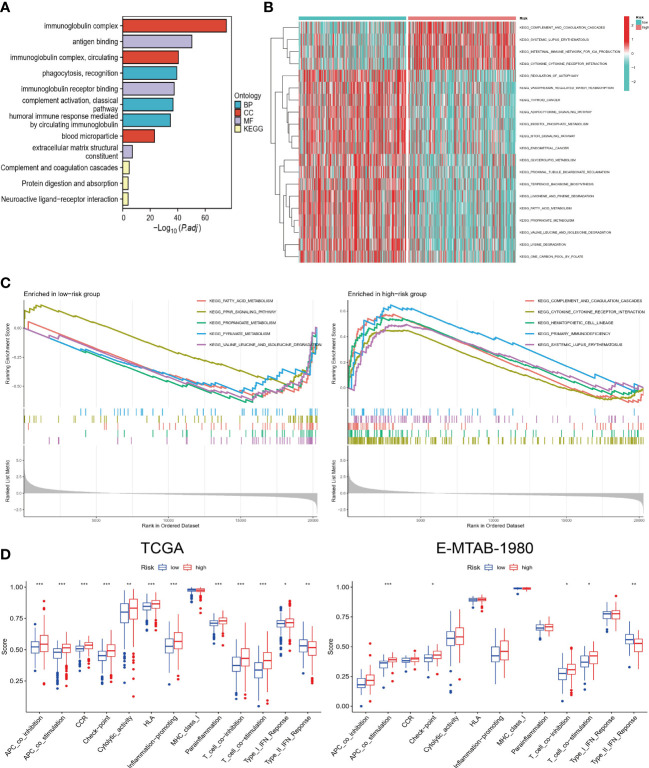
Functional and pathway enrichment analysis. **(A)** GO and KEGG enrichment results of DEGs. **(B)** GSVA in high and low-risk groups. **(C)** GSEA of high and low-risk groups. **(D)** Comparison of differences in immune-related functions between high and low-risk groups in TCGA and E-MTAB-1980 cohorts. “*”<0.5, "**" <0.01, and "***"<0.001.

The results of GO and KEGG analysis showed that the differential genes between high and low-risk groups were mainly enriched in immune-related biological functions and pathways, such as immunoglobulin complex and antigen binding (**Figure 5A**). It appears that pathway enrichment distribution in the two groups differs significantly according to the GSVA results, with the high-risk group mainly enriched in immune-related pathways and the low-risk group enriched in metabolism-related pathways (**Figure 5B**). As a result of GSEA, the top 5 most significantly enriched pathways are shown. The immune-related pathways that were significantly enriched in the high-risk group included cytokine-cytokine receptor interaction and primary immunodeficiency (**Figure 5C**). Furthermore, we calculated the immune function score for each sample. High-risk individuals scored higher on all immune-related functions except type II IFN response (**Figure 5D**). The same trend was maintained in the analysis of the validation cohort. Hence, combining the results of these analyses, it is reasonable to assume that risk scores are closely related to the TME of ccRCC and could potentially guide immunotherapy.

### Immunotherapy response and drug sensitivity

**Figure 6:**
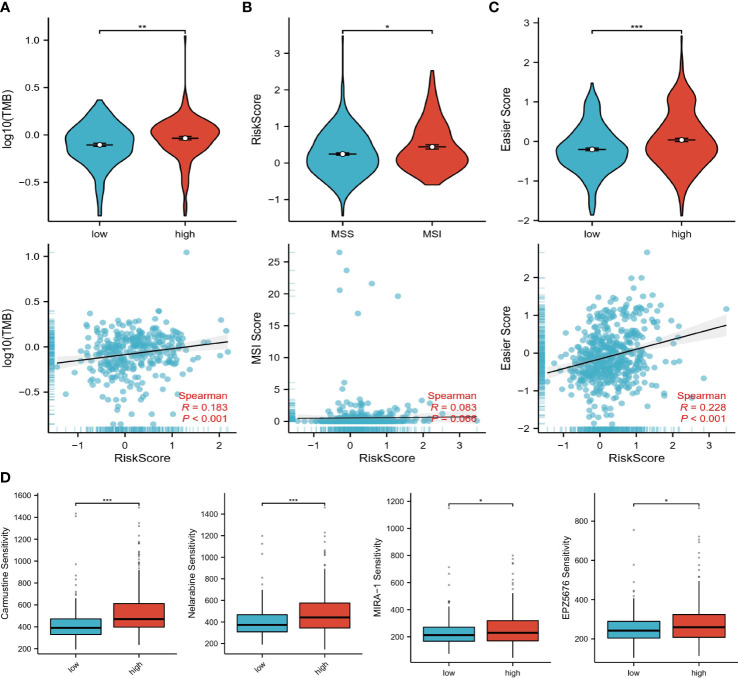
Immunotherapy and drug prediction. **(A)** TMB levels in high and low-risk groups, correlation of risk scores with TMB. **(B)** Levels of risk scores in MSI and MSS groups, correlation of risk scores with MSI scores. **(C)** Levels of easier scores in high and low-risk groups, correlation of risk scores with easier scores. **(D)** Differences in sensitivity to different drugs in high and low-risk groups. “*”<0.5, "**" <0.01, and "***"<0.001

Further exploring immunotherapy response, we compared the difference between the two risk groups according to TMB, MSI, and easier scores, with higher values predicting a greater likelihood of benefiting from immunotherapy. We observed that patients in the high-risk group had higher TMB and easier scores, and that risk scores changed positively with TMB (**Figures 6A–C**). A higher risk score was also observed in the MSI group (**Figure 6B**). Consequently, immunotherapy was more likely to succeed in high-risk patients, resulting in an improved outcome. Subsequently, we calculated the sensitivity of each sample to different drugs and then compared the differences in sensitivity of the top 5 drugs in two risk groups by ranking the mean of the sensitivities. Among those at high risk, Carmustine, Nelarabine, MIRA-1, and EPZ5676 appeared to be more sensitive. Perhaps, the combination of these drugs with immune checkpoint inhibitors could lead to surprising therapeutic effects, which provides new ideas for future prospective study designs.

### Key model genes affecting risk scores

**Figure 7:**
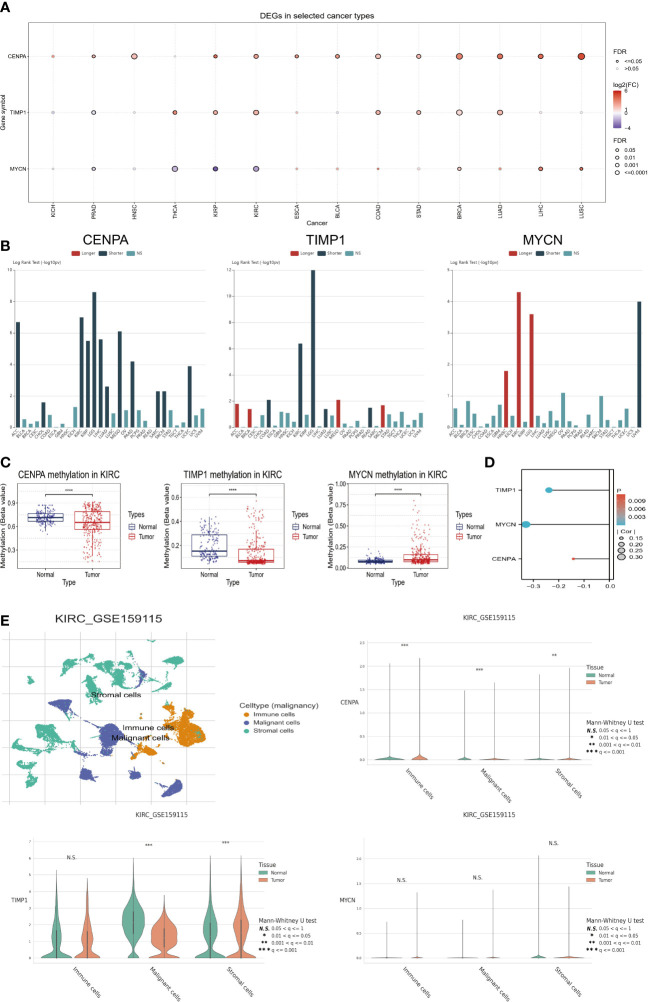
Comprehensive analysis of key model genes. **(A)** Pan-cancer analysis of gene expression levels. **(B)** Pan-cancer survival analysis. **(C)** Methylation differences between tumor and normal tissues. **(D)** Relationship between methylation levels and gene expression. **(E)** Single-cell analysis of key model genes. “****” <0.0001.

We in-depth explored the three genes with the highest risk coefficient, including *CENPA, TIMP1*, and *MYCN*. Firstly, **Figure 7A** demonstrates their expression levels in pan-cancer. *CENPA* and TIMP1 are highly expressed in the tumor tissue of most cancers, while *MYCN* is usually lowly expressed. We also found that they were closely associated with OS in several cancer types. As with *CENPA*, *TIMP1* was associated with poor prognosis in most cancers. *MYCN* was mainly associated with good prognosis, especially in ccRCC, where higher *MYCN* expression was associated with longer OS in patients (**Figure 7B**). We also evaluated the methylation status of the three genes in tumors and normal tissues. The methylation levels of *CENPA* and *TIMP1* were downregulated in tumor tissues, while *MYCN*, in contrast to them, had elevated methylation levels (**Figure 7C**). Moreover, their expression levels were negatively correlated with the methylation levels (**Figure 7D**), which suggests that methylation could be responsible for their differential expression levels. Using single-cell sequencing data from GSE159115, we further investigated *CENPA, TIMP1*, and *MYCN* expression levels in different cell types. As shown in **Figure 7E**, all cells can be broadly classified into three types, including immune cells, malignant cells, and stromal cells. Compared with normal tissues, *CENPA* expression in tumor tissues was elevated in all three cell types; *TIMP1* was elevated in stromal cells and decreased in malignant cells; *MYCN* expression levels were not significantly different in different cell types.

**Figure 8:**
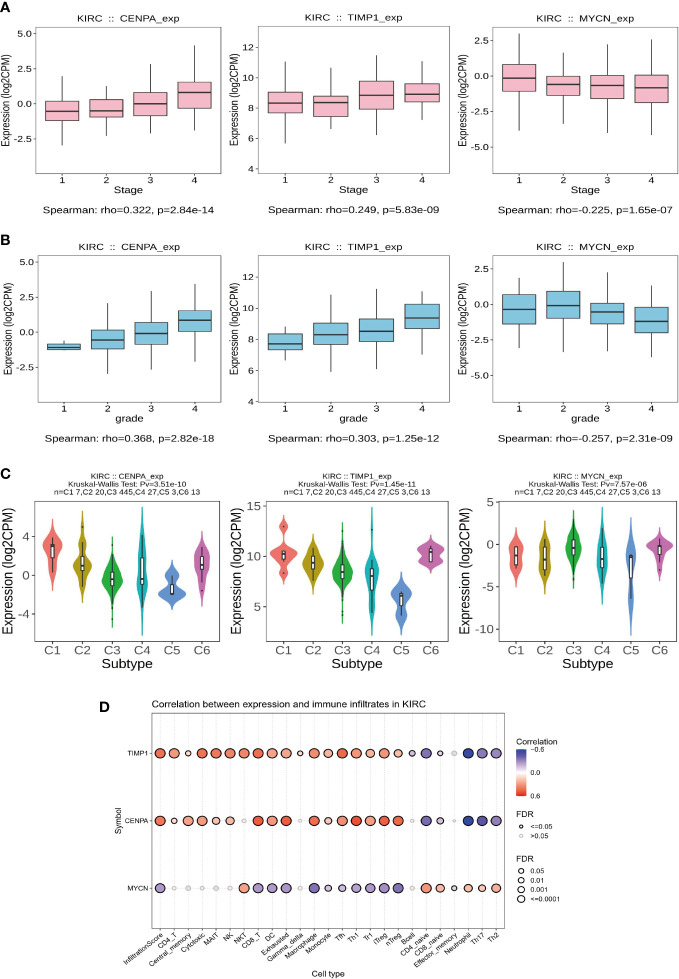
Clinical features and immunological correlates. **(A)** Relationship between gene expression levels and stage. **(B)** Relationship between gene expression levels and grade. **(C)** Key model genes classify ccRCC into 6 immune subtypes. **(D)** The relationship between gene expression levels and the abundance of immune cell infiltration.

Subsequently, we analyzed the relationship of *CENPA, TIMP1*, and *MYCN* with the clinical features and TME of ccRCC. To begin with, the expression levels of *CENPA* and *TIMP1* increased with the tumor stage, while *MYCN* did the opposite (**Figure 8A**). A similar trend was observed in tumor grade, where the expression levels of *CENPA* and *TIMP1* were positively correlated with grade and that of *MYCN* was negatively correlated (**Figure 8B**). Based on these genes, ccRCC can be classified into different immune subtypes (**Figure 8C**), suggesting a close relationship between them and the tumor’s TME. This is further confirmed by the analysis presented in **Figure 8D**, where *CENPA* and *TIMP1* were positively correlated with the infiltration level of most immune cells, and *MYCN* showed the opposite trend (**Figure 8D**). Therefore, we believe that *CENPA*, *TIMP1*, and *MYCN* are all therapeutic targets with significant potential in ccRCC.

### Nomogram

**Figure 9:**
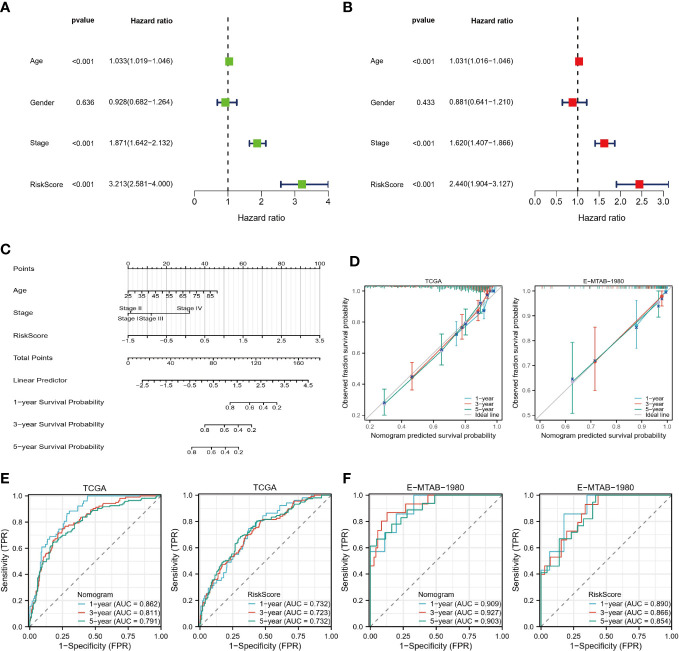
Construction of Nomogram. **(A, B)** Univariate and multivariate COX regression analysis. **(C)** Nomograms predict 1-, 3-, and 5-year survival probabilities. **(D)** Calibration plots for the TCGA and E-MTAB-1980 cohorts. **(E, F)** ROC curves of nomograms and risk scores in the TCGA and E-MTAB-1980 cohorts.

An independent predictor of OS in patients with ccRCC was age, stage, and risk score, as identified by univariate and multivariate COX regression analyses (**Figures 9A, B**). A nomogram was developed to predict survival probability in patients with ccRCC at 1, 3, and 5 years (**Figure 9C**). As seen in the calibration plots (**Figure 9D**), the predicted probabilities were almost in a straight line with the actual possibilities for both the TCGA and E-MTAB-1980 cohorts, indicating that the prediction results of the Nomogram were very accurate. Additionally, the results of ROC analysis also show that our nomogram has a high predictive value. The one-year Area Under Curve (AUC) of the nomogram in the TCGA cohort equals 0.862 (one-year AUC for risk score=0.732). The 1-year AUC of the nomogram for the E-MTAB-1980 cohort is 0.909 (0.890 for the risk score). In other words, our nomograms provide accurate prognostic information to patients and clinicians.

### Expression validation of key model genes

**Figure 10:**
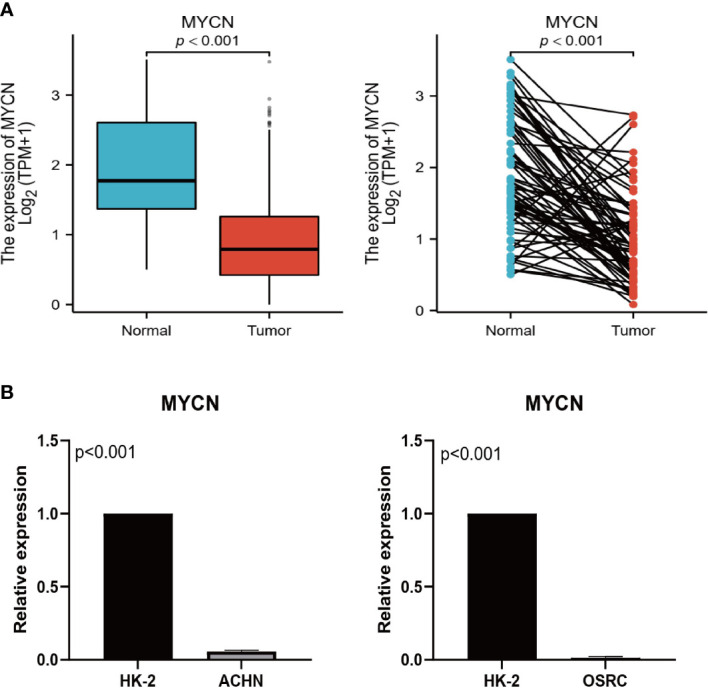
Expression validation of MYCN. **(A)** Expression levels of MYCN in TCGA unpaired and paired samples. **(B)** Expression levels of MYCN in HK-2, ACHN, and OSRC cell lines.

Wang (25) and Shou (26) have confirmed through detailed *in vitro* experiments that *CENPA* and *TIMP1* are highly expressed in ccRCC tumor tissues and promote tumor progression through different mechanisms. As a result, we assessed *MYCN* expression levels in tumors and normal tissues using RT-qPCR. Primer sequence of *MYCN*: F: CAGGCAAGACAGCAGCA; R: ATGTGCAAAGTGGCAGTGA. The *MYCN* expression in ACHN and OSRC cell lines was significantly lower than that in HK-2 normal cells (**Figure 10B**), which was in perfect agreement with the results of TCGA-KIRC analysis (**Figure 10A**).

## Discussion

As the most common pathological type of RCC, ccRCC is highly immunogenic and heterogeneous. In other words, the TME may be dramatically different for different ccRCC, and this may account for the large differences in response rates to immune checkpoint inhibitors in different patients (27). Furthermore, it is due to such a highly heterogeneous TME that metabolites associated with tumor biological behavior are also increased (28). In numerous studies, metabolic reprogramming has been shown to occur during the development of ccRCC, which controls the tumor’s energetic and biosynthetic metabolisms (29). p M Herst’s study also pointed out that MMR is an important factor in determining tumor fate (30). Furthermore, Bianca’s study showed that MMR can induce the onset of ICD and thus exert tumor-killing effects (31). Therefore, an in-depth investigation of the significance of MMR and ICD in the TME of ccRCC is highly promising. As far as we know, our study is the first to combine these factors to reveal ccRCC prognostic factors and TME characteristics.

Our study integrated MMR and ICD-related genes and identified 140 genes with a significantly different expression between tumor and normal tissues. From these, 12 genes most associated with OS were then screened for use in constructing risk models. PCA and K-M survival analysis showed that risk grouping could distinguish different ccRCC patients well. Subsequently, we analyzed the differences in the TME, TMB, MSI, and functional clustering between the two risk groups to help identify ccRCC patients who have a greater chance of benefitting from immunotherapy. In addition, our comprehensive analysis of key model genes showed that CENPA, TIMP1, and MYCN are all therapeutic targets with significant potential. In the end, we constructed a nomogram with strong predictive performance to predict the survival probability of patients, which makes our study more clinically relevant.

According to our findings, in the high-risk group, CD8 T cells and TFH were more prevalent, as well as higher immune scores. this may seem contradictory, as generally, a high level of immune cell infiltration would inhibit tumor progression and thus improve patient prognosis. Perhaps the heterogeneity between different tumors causes ccRCC to differ from other immunotherapy-responsive solid tumors (32). We also found higher expression of the immune checkpoints PD-1 and CTLA4 in the high-risk group. The study by Pramod et al. points to the abundance of immune cell infiltration in the TME and the expression level of immune checkpoint genes as potential biomarkers for predicting immunotherapeutic response (33), and that abundant CD8 T-cell infiltration and immune checkpoint overexpression are associated with an excellent immunotherapeutic response. High-risk individuals also exhibited a greater abundance of immune-related pathways and functions, and the higher levels of TMB, MSI, and easier scores further validated our findings (22, 23). A risk model associated with the immune landscape of ccRCC was constructed by Zhuo et al. based on lactate metabolism-related genes, and they obtained similar results, inhibitors of immune checkpoints are more likely to work for patients with high-risk conditions (14). However, Wu’s findings were opposed to ours (34). Although they found that the high-risk group also had higher immune checkpoint expression and CD8 T-cell infiltration, they used the Tumor Immune Dysfunction and Exclusion (TIDE) scores to determine the response to immunotherapy in two risk groups. Perhaps, this is the reason for the very different results.

Subsequently, we analyzed the prognostic features and immune correlations of three key genes determining risk scores in ccRCC from multiple perspectives. Pan-cancer analysis revealed that Centromere Protein A (*CENPA*) and Metallopeptidase Inhibitor 1 (*TIMP1*) were highly expressed in most cancers and significantly associated with a poorer prognosis, also in ccRCC. *CENPA* overexpression enhanced ccRCC proliferation and metastasis by activating the Wnt/Kip1 pathway, according to Wang et al. (25). The protein encoded by *TIMP1* is not only a natural inhibitor of matrix metalloproteinases but is also closely associated with cell proliferation and apoptosis in several cell types (35). Furthermore, *in vitro* experiments and bioinformatics analysis conducted by Shou’s study revealed that *TIMP1* promotes RCC progression *via* the epithelial-mesenchymal transition signaling pathway (26). However, they do not elaborate on what roles *CENPA* and *TIMP1* play in the TME of ccRCC. A significant correlation was found between *CENPA* and *TIMP1* expression in tumor tissues and immune cell infiltration, and as expression increased, most immune cell infiltration levels increased. Moreover, ccRCC could be classified into different immune subtypes based on the expression of *CENPA* and *TIMP1*. This all suggests that *CENPA* and *TIMP1* are highly promising biomarkers and therapeutic targets. Usually, *MYCN* is associated with poor prognosis because it is part of the *MYC* family. In particular, it plays a key role in pediatric neuroblastoma, where it can promote tumor cell growth and appreciation based on metabolic reprogramming (36). Nevertheless, our pan-cancer analysis showed that *MYCN* appears to be a protective factor for ccRCC and is much less expressed in renal tumor tissues. Moreover, *MYCN* was closely related to the immune subtype of ccRCC and the infiltration abundance of immune cells. However, no studies have been conducted to analyze in depth the potential mechanisms by which *MYC*N affects the prognosis of ccRCC, so we do not yet know why *MYCN* plays a different role in ccRCC than in other cancers. In other words, our study identified a novel and promising biomarker for ccRCC, but more reliable *in vitro* and *in vivo* experiments are needed to validate our findings.

In conclusion, our study constructs a 12-gene risk model based on MMR and ICD-related genes, which can accurately distinguish patients at different risks and guide patients for immunotherapy and targeted therapies. Furthermore, a nomogram based on risk scores and clinical variables can also accurately predict a patient’s OS, progress-free survival (PFS), and disease-specific survival (DSS) for 1, 3, and 5 years (**Supplementary documents Figure1**), and have high clinical application value. However, there are some shortcomings in our study. At first, our study was mainly a retrospective analysis based on public databases, and future high-quality prospective studies are needed to enhance the credibility of the conclusions. Secondly, we did not reveal the specific mechanism of action of key model genes by experimental means.

## Conclusion

In this study, we revealed the TME and prognostic features of ccRCC from the perspective of mitochondrial metabolism and programmed cell death and established a new risk model and nomogram to distinguish patients with different molecular and clinical characteristics, which can be used as a reliable tool to predict the prognosis and immunotherapeutic response of ccRCC patients. Furthermore, the identification of the key molecule MYCN will facilitate the development of effective targeted drugs for ccRCC from the perspective of targeting mitochondrial metabolism. To conclude, our study provides both new insights into the regulatory mechanisms of MMR in ccRCC and assists clinicians in making rational decisions.

## Data availability statement

The original contributions presented in the study are included in the article/**Supplementary Material**, further inquiries can be directed to the corresponding authors.

## Ethics statement

It was approved by Nanchang University’s First Affiliated Hospital’s Research Ethics Committee (Nanchang, China).

## Author contributions

LY: Data curation, formal analysis, writing - original draft, writing - review & editing. SL, XL, and WD: Data curation, formal analysis. BF, JX, and WL: Conceptualized research, writing - review & editing. They all agreed to publish. All authors contributed to the article and approved the submitted version.

## Glossary

**Table:**

TMB	tumor microenvironment
RCC	renal cell carcinoma
ccRCC	clear cell renal cell carcinoma
MMR	mitochondrial metabolic reprogramming
ICD	immunogenic cell death
TCGA	the Cancer Genome Atlas
WGCNA	weighted gene co-expression network analysis
TOM	topological overlap matrix
TGs	target genes
DEGs	differentially expressed genes
FC	fold change
K-M	Kaplan-Meier
TMB	tumor mutational load
GO	Gene Ontology
KEGG	Kyoto Encyclopedia of Genes and Genomes
OS	overall survival
LASSO	least absolute shrinkage and selector operation analysis
PCA	principal component analysis
GSEA	gene set enrichment analysis
GSVA	gene set variation analysis
MSI	microsatellite instability
ROC	receiver operating characteristic
RT-qPCR	real-time fluorescence quantitative polymerase chain reaction
TFH	T cell follicular helper
PFS	Progress-free survival
DSS	Disease-specific survival
