# Supplementary material for: Mitochondrial metabolic reprogramming-mediated immunogenic cell death reveals immune and prognostic features of clear cell renal cell carcinoma
Source: Front Oncol. 2023 May 5;13:1146657. doi: 10.3389/fonc.2023.1146657 (PMC10196130; doi:10.3389/fonc.2023.1146657)
Supplement: Supplementary file 1 [file DataSheet_1.docx]

Table 1 Baseline characteristics of the high and low-risk groups in the TCGA cohort

| Characteristics | low risk | high risk | P value |
| --- | --- | --- | --- |
| n | 266 | 263 |  |
| Age, median (IQR) | 60 (50.25, 69) | 62 (53, 70) | 0.153 |
| Sex, n (%) |  |  | < 0.001 |
| Female | 114 (21.6%) | 72 (13.6%) |  |
| Male | 152 (28.7%) | 191 (36.1%) |  |
| Grade, n (%) |  |  | < 0.001 |
| G1 | 10 (1.9%) | 4 (0.8%) |  |
| G2 | 148 (28.4%) | 78 (15%) |  |
| G3 | 91 (17.5%) | 114 (21.9%) |  |
| G4 | 11 (2.1%) | 65 (12.5%) |  |
| T-stage, n (%) |  |  | < 0.001 |
| T1 | 174 (32.9%) | 97 (18.3%) |  |
| T2 | 30 (5.7%) | 39 (7.4%) |  |
| T3 | 61 (11.5%) | 117 (22.1%) |  |
| T4 | 1 (0.2%) | 10 (1.9%) |  |
| N-stage, n (%) |  |  | 0.009 |
| N0 | 109 (43.1%) | 128 (50.6%) |  |
| N1 | 2 (0.8%) | 14 (5.5%) |  |
| M-stage, n (%) |  |  | < 0.001 |
| M0 | 232 (46.7%) | 186 (37.4%) |  |
| M1 | 18 (3.6%) | 61 (12.3%) |  |
| Stage, n (%) |  |  | < 0.001 |
| Stage I | 172 (32.7%) | 93 (17.7%) |  |
| Stage III | 46 (8.7%) | 75 (14.3%) |  |
| Stage IV | 19 (3.6%) | 64 (12.2%) |  |
| Stage II | 29 (5.5%) | 28 (5.3%) |  |
| OS (days), median (IQR) | 1369 (686, 2074.8) | 1034 (438, 1777) | 0.002 |
| Status, n (%) |  |  | < 0.001 |
| Alive | 219 (41.4%) | 138 (26.1%) |  |
| Dead | 47 (8.9%) | 125 (23.6%) |  |

Table 2 Baseline characteristics of the high and low-risk group in the E-MTAB-1980 cohort

| Characteristics | low risk | high risk | P value |
| --- | --- | --- | --- |
| n | 51 | 50 |  |
| Age, mean ± sd | 60.392 ± 11.844 | 66.62 ± 10.333 | 0.006 |
| Sex, n (%) |  |  | 0.178 |
| Female | 15 (14.9%) | 9 (8.9%) |  |
| Male | 36 (35.6%) | 41 (40.6%) |  |
| Grade, n (%) |  |  | 0.107 |
| 1 | 9 (9.1%) | 4 (4%) |  |
| 2 | 33 (33.3%) | 26 (26.3%) |  |
| 3 | 8 (8.1%) | 14 (14.1%) |  |
| 4 | 1 (1%) | 4 (4%) |  |
| T-stage, n (%) |  |  | 0.023 |
| T1 | 41 (40.6%) | 27 (26.7%) |  |
| T2 | 4 (4%) | 7 (6.9%) |  |
| T3 | 6 (5.9%) | 15 (14.9%) |  |
| T4 | 0 (0%) | 1 (1%) |  |
| N-stage, n (%) |  |  | 0.112 |
| N0 | 50 (49.5%) | 44 (43.6%) |  |
| N2 | 1 (1%) | 3 (3%) |  |
| N1 | 0 (0%) | 3 (3%) |  |
| M-stage, n (%) |  |  | 0.515 |
| M0 | 46 (45.5%) | 43 (42.6%) |  |
| M1 | 5 (5%) | 7 (6.9%) |  |
| Stage, n (%) |  |  | 0.083 |
| Stage I | 39 (38.6%) | 27 (26.7%) |  |
| Stage IV | 5 (5%) | 7 (6.9%) |  |
| Stage II | 4 (4%) | 6 (5.9%) |  |
| Stage III | 3 (3%) | 10 (9.9%) |  |
| OS (days), median (IQR) | 1860 (1275, 2625) | 1275 (592.5, 2077.5) | 0.014 |
| Status, n (%) |  |  | < 0.001 |
| Alive | 48 (47.5%) | 30 (29.7%) |  |
| Dead | 3 (3%) | 20 (19.8%) |  |

Supplementary documents Fig1


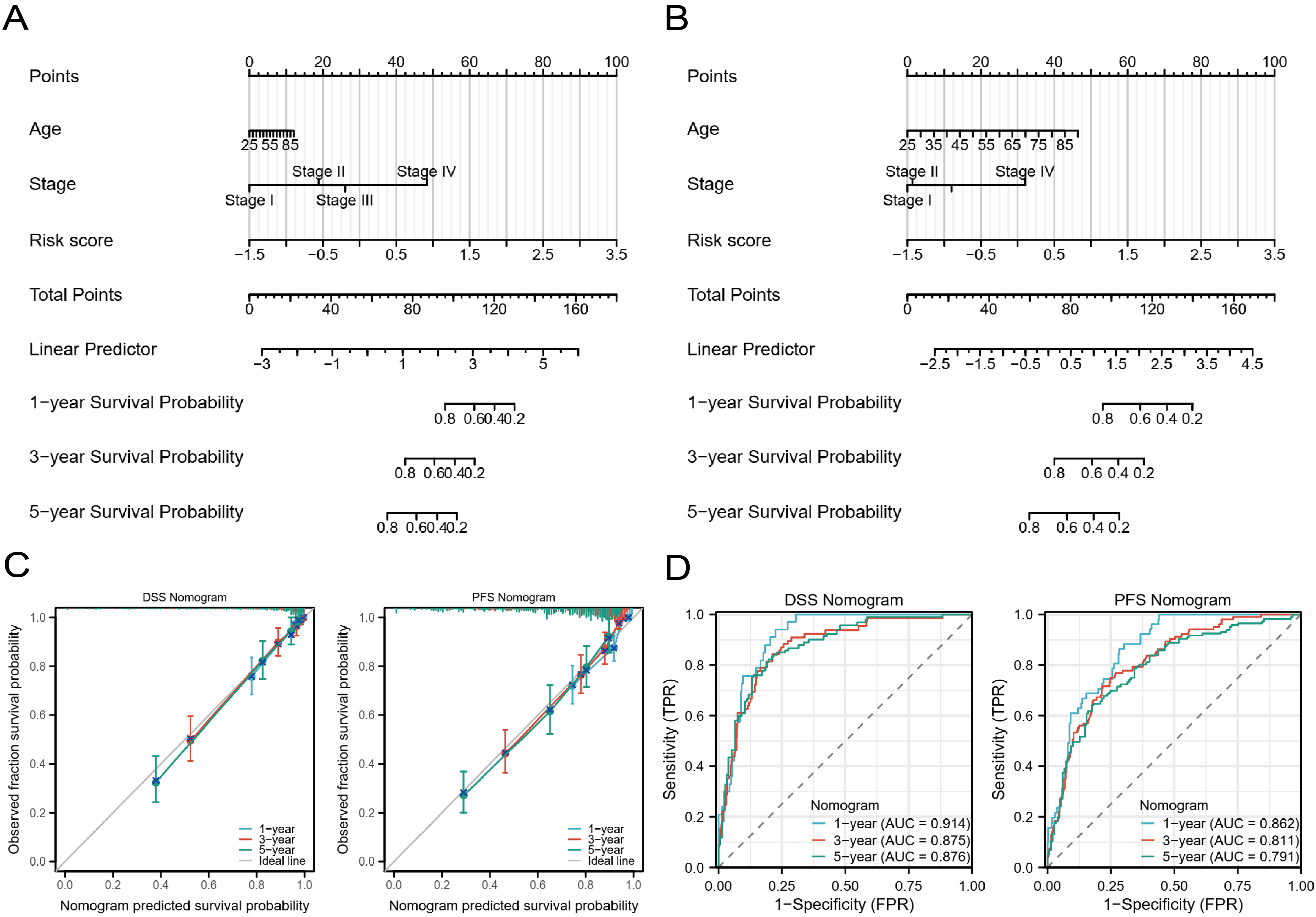


A: Nomograms predict 1-, 3-, and 5-year DSS probabilities. B: Nomograms predict 1-, 3-, and 5-year PFS probabilities. C: Calibration plots for the Nomograms. D: ROC curves of nomograms in the DSS and PFS.
